# A Pilot Colorectal Cancer Study Using Fecal Occult Blood Tests and Colonoscopy to Identify the Weaknesses of the Romanian Public Healthcare System before Implementing National Screening

**DOI:** 10.3390/ijerph20032531

**Published:** 2023-01-31

**Authors:** Linda-Nicoleta Bărbulescu, Stelian-Ștefăniță Mogoantă, Lucian-Florentin Bărbulescu, Constantin Kamal, Didi-Liliana Popa, Radu-Teodoru Popa

**Affiliations:** 1 Doctoral School, University of Medicine and Pharmacy of Craiova, 200349 Craiova, Romania; 2 Cabinet Medical Dr Profir I Mirela SRL, 200145 Craiova, Romania; 3 Department of Surgery, University of Medicine and Pharmacy of Craiova, 200349 Craiova, Romania; 4 Department III of Surgery, University Emergency County Hospital, 200642 Craiova, Romania; 5 Department of Computers and Information Technology, University of Craiova, 200585 Craiova, Romania; 6 Department of Family Medicine, University of Medicine and Pharmacy of Craiova, 200349 Craiova, Romania; 7 Doctoral School “Constantin Belea”, University of Craiova, 200585 Craiova, Romania

**Keywords:** colorectal cancer screening, family medicine, primary health care, preventive medicine, health education

## Abstract

The objective of this study is to investigate the feasibility of colorectal cancer (CRC) screening in the absence of a national screening program using the resources provided by the Romanian healthcare system. Study participants were recruited from adult patients (over 18 years old) registered with a general practitioner from an urban area over a period of 3 years (October 2019 to September 2022). Patients were recruited when they came for a consult at their family physician’s office. The study excluded patients with a medical history of colorectal cancer. Written consent was obtained from the patients who agreed to participate. Patients who agreed to participate were recommended to undergo a fecal occult blood test (FOBT). For those with a positive FOBT result, a colonoscopy was recommended. The study identified a need and willingness of patients to participate in CRC screening when they were informed about it, especially when it involved a noninvasive test such as FOB. We did not anticipate the refusal to perform FOBT in the public healthcare system because the recommendation was made by a GP. We identified a deficit of specialists that can perform colonoscopies in the public healthcare system, insufficient health education, and a lack of dedicated pathways for screening.

## 1. Introduction

The pilot screening program aims to outline the number and type of problems that may occur when a colorectal cancer screening program is implemented in Romania. Some of the issues are known from other countries’ colorectal screening programs. Bearing in mind that every country has unique features that can influence the outcome in one way or another, we wanted to identify the main obstacles that can occur in the implementation of a nationwide colorectal cancer screening program.

Colorectal cancer (CRC) is a disease ranked third from the top of global cancer incidence in 2020, after breast cancer in women and lung cancer. The mortality from colorectal cancer takes second place as the leading cause of cancer-related deaths after lung cancer on a global level [1,2]. According to GLOBOCAN 2020, colorectal cancer was in first place in Romania in the number of newly diagnosed cases in 2020 in both sexes and at all ages (13.1%) [3]. According to the Romanian National Institute of Public Health, the incidence of CRC was 12.7% in 2020, with 6670 new cases [4]. Mortality by CRC ranked second to mortality by lung cancer in overall cancer deaths in 2020, with 4109 deaths (18.56 per 100,000 people) [5]. The mortality rate of treatable causes is the highest in the EU and is more than twice the average of EU countries [6]. The World Cancer Research Fund International places Romania ninth in the world in total global colorectal cancer mortality in 2020, with an age-standardized rate per 100,000 of 14.7 [7].

Romania and Bulgaria are the only EU member states without a colorectal cancer screening program. This is most likely the primary reason why the utilization of colorectal cancer screening tests in Romania is very low (6.3%) [8,9]. Another reason may be the fact that family doctors, or GPs as they are known in Romania, do not have training in CRC screening. They are given access to different training programs but only if they wish to do so. This is why GPs are unaccustomed to performing CRC screening. Many of them are unaware that the FOBT is a test they can recommend.

Colorectal cancer screening is essential to identifying precancerous lesions and thus lowering the mortality rate. The costs of screening are lower than the costs of trying to treat the cancer. There are several modifiable CRC risk factors among Romanians: obesity and physical inactivity, consumption of red and processed meat, smoking, alcohol consumption, medications, and diabetes. This gives reasonable justification to speed up the screening process law [10]. Early screening has been demonstrated to improve clinical outcomes for CRC. Assessing patterns in CRC screening utilization is critical to guiding policy and implementing programs for CRC prevention and control [11].

There are a multitude of factors that contribute to the high number of advanced colorectal cancer patients that seek medical attention from their family doctor or other specialties such as internal medicine, gastroenterology, and general surgery. Among these factors, the most significant ones are the lack of screening programs financed by the government, poor access to medical care, and the lack of health education among the general population.

A study identified fifteen guidelines for CRC screening. Six guidelines were published in North America, four in Europe, four in Asia, and one by the World Gastroenterology Organization. In these guidelines, it is recommended to start CRC screening at 50 years old and continue until 75 years old using fecal occult blood tests annually or biennially (mainly using the fecal immunochemical test (FIT)), colonoscopy (every 10 years), or flexible sigmoidoscopy (every 5 years) [12]. These recommendations can be successfully used in the Romanian CRC screening program.

CRC screening should be lowered to 45 years of age, as the American Cancer Society recommended in 2018 [13,14]. The U.S. Preventive Service Task Force states that age is one of the most influential risk factors for colorectal cancer. Incidence rates increase with age, and nearly 94% of new cases of colorectal cancer occur in adults 45 years or older. It is estimated that 10.5% of newly diagnosed colorectal cancer cases occur in persons younger than 50 years old. The incidence of colorectal cancer (specifically adenocarcinoma) in adults aged 40 to 49 has increased by almost 15% from 2000–2002 to 2014–2016 [15].

Several methods are used to screen for CRC, including blood stool tests, sigmoidoscopy, and colonoscopy as recommended in the guidelines [11,12]. Currently, little information is available on the effectiveness of organized colorectal cancer (CRC) screening on screening uptake, incidence, and mortality in community-based populations [16]. Several randomized population-based studies have shown that screening for colorectal cancer (CRC) by using fecal occult blood tests (FOBTs) can reduce CRC mortality [17,18,19,20,21].

## 2. Materials and Methods

The study was performed in Doctor Linda-Nicoleta Bărbulescu’s practice, an average-sized family medicine practice in an urban area that had 1490 patients registered (666 men and 824 women), of which 1200 were insured and 290 were uninsured.

The pilot screening program was initiated in October 2019. Initially, we wanted to develop the program for two years, but due to the COVID-19 pandemic, we decided to extend it to three years. We proposed a pilot study in an adult population (only those that were registered with a family physician or general practitioner trained in CRC screening) in an urban area. Exclusion factors were a history of colorectal cancer and patients without insurance in the Romanian public healthcare system. We tried to implement a CRC screening program using the resources that were already provided by the Romanian public healthcare system without incurring any costs for the patients in the absence of a national screening program.

Of all patients, 98.5% were Caucasian and the remainder were Asian or African (most of these were foreign students). Of the insured patients, 995 were more than 18 years old (82.91%), divided as follows: 203 patients between 18 and 39 (16.91%), 114 patients between 40 and 44 (9.5%), 75 patients between 45 and 49 (6.25%), 385 patients between 50 and 69 (32.08%), 91 patients between 70 and 74 (7.58%), 55 patients between 75 and 80 (4.58%), and 72 patients between 81 and 96 (6%) years old.

**Figure 1:**
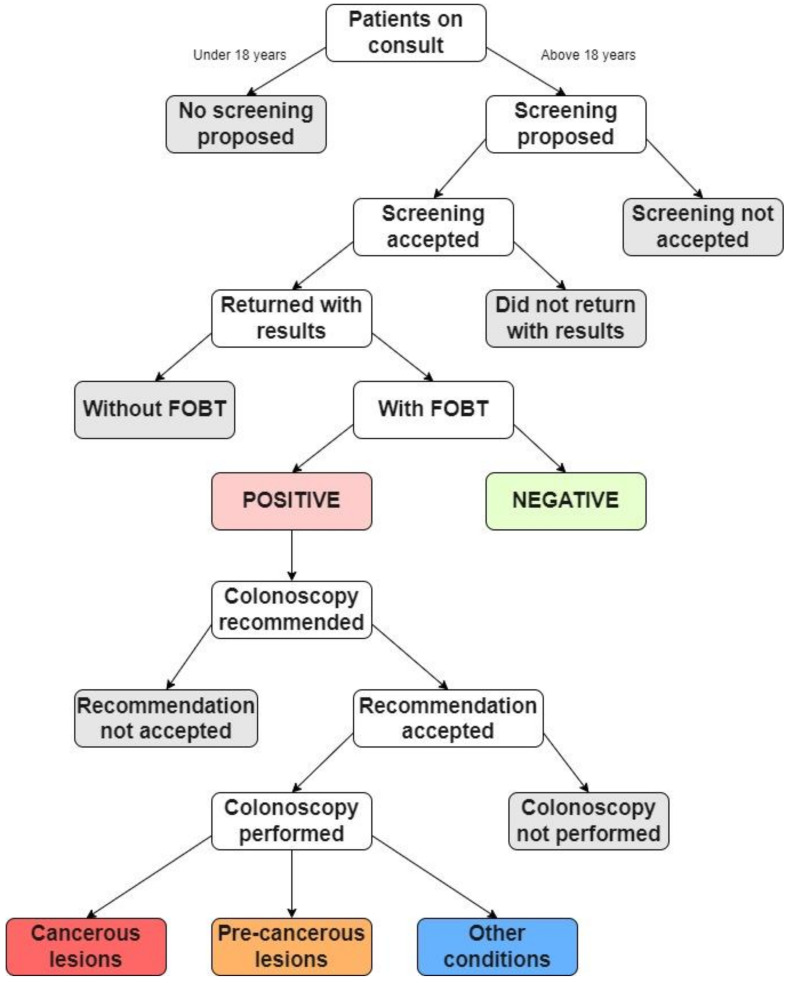
Flow chart summarizing the study’s activities.

For the study, every adult patient (over 18 years old) who came for a consultation at the family medicine practice for 3 years (1 October 2019 to 30 September 2022), regardless of their chief complaint, was asked to participate in colorectal cancer screening. Those who agreed to participate in the study were recommended a fecal occult blood test (FOBT). Two FOBT methods were used: the high-sensitivity guaiac fecal occult blood test (gFOBT) and the fecal immunochemical test (FIT) [22]. Either can be used in the laboratory; thus, all patients received dietary instructions. The dietary instructions were given due to the sensitivity of gFOBT to certain foods, supplements, and anti-inflammatory medications, which can lead to false positives. Not every patient that agreed to the screening returned a positive FOBT result. The patients that returned a positive FOBT result were recommended for further evaluation by another doctor (internist, gastroenterologist, or surgeon) and followed up with a colonoscopy. Finally, the patients that had a colonoscopy returned a result that was either a cancerous lesion, a precancerous lesion, or another condition (internal or external hemorrhoids). Figure 1 presents a diagram of the study’s activities.

## 3. Results

During the first year of screening (October 2019 to September 2020), from a total of 97 FOB tests recommended to unique patients, only 47 returned an FOBT result (48.45%). Of the 47 FOBT results returned, only 8 patients (17.02%) had a positive result. Two patients with positive FOBT results decided to retake the test due to noncompliance with the dietary instructions. These FOBT results were negative. Since then, they have undertaken an FOBT annually and the results have been negative. In conclusion, in the first year, only six patients remained with a positive FOB test result (12.76%).

During the second year of screening (October 2020 to September 2021), from a total of 40 FOB tests recommended to unique patients, only 21 returned an FOBT result (52.5%). Of the 21 FOBT results returned, only 6 patients (28.57%) had a positive result. One patient with a positive FOBT result decided to retake the test due to noncompliance with the dietary instructions. The second FOBT result was negative. One patient had a positive result the year before, and it was the second time the FOB test was positive. In conclusion, in the second year, only four patients had a positive FOB test result (19.04%).

During the third year of screening (October 2021 to September 2022), from a total of 31 FOB tests recommended to unique patients, only 21 returned an FOBT result (61.76%). Of the 21 FOBT results returned, only 2 patients (9.52%) had a positive result.

**Figure 2:**
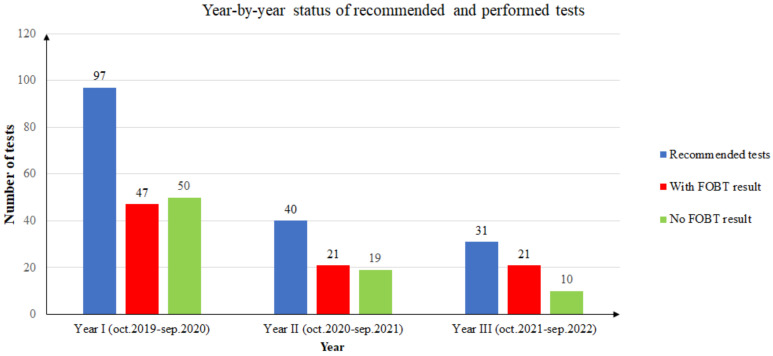
Year-by-year status of recommended and performed tests.

It should be noted that the year-by-year statistics use unique patients. If a patient took multiple tests in one year, the person was counted only once, and a positive result superseded any other result for the patient in that year. If a patient took the test in different years, the results were counted in each year’s statistics. Figure 2 presents the summarized three-year distribution of recommended and performed tests.

**Figure 3:**
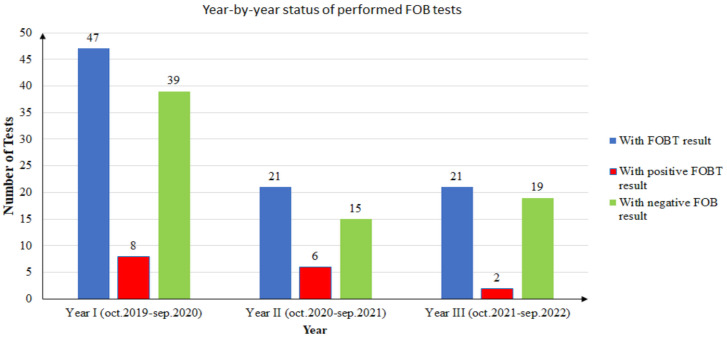
Year-by-year status of performed FOB tests.

During the study, there were 15 unique patients with positive FOBT results. One patient did the test twice in two consecutive years, and the results were positive each time. In conclusion, there were 16 positive FOBT results from 15 unique patients. The year-by-year distribution of positive and negative results is presented in Figure 3.

During the 3 years, only 144 unique patients agreed to participate in the study. Out of the 144 receivers of the FOBT recommendation, 68 participants did not return test results. A total of 76 tests were performed, resulting in an FOBT uptake rate (UR) of 52.77%. There were 15 positive tests, for a positivity rate of 19.73%. Table 1 presents the distribution of the uptake rate at CRC screening by age group.

**Figure 4:**
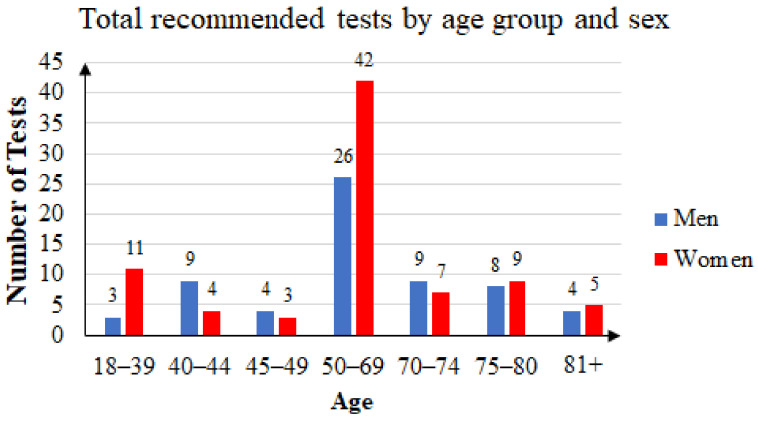
Three years’ statistics: total of recommended tests by age group and sex.

**Figure 5:**
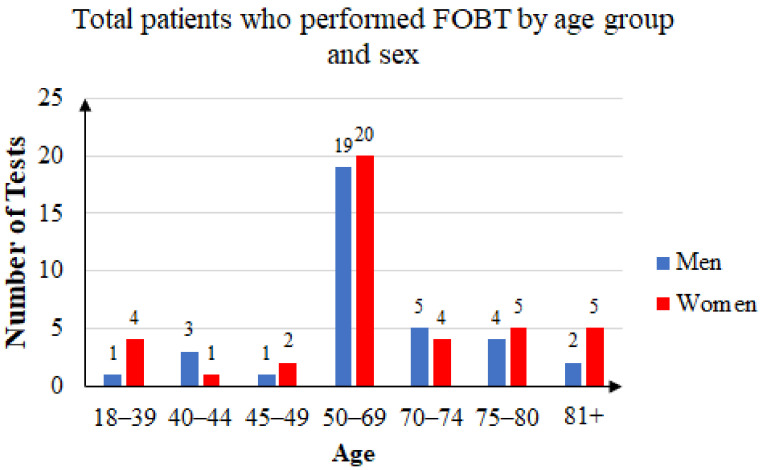
Three years’ statistics: total patients who performed FOB tests by age group and sex.

Some of the patients took the FOB test two or three times. Of the 144 unique patients that agreed to participate in the study, 63 were men and 81 were women. The mean age was 60.41 years: 59.76 years for women and 61.25 years for men. Female subjects accounted for 56.25% of the total. Figure 4 summarizes the age and sex distribution of patients who were recommended FOB tests, and Figure 5 summarizes that of the patients who returned with FOBT results.

**Figure 6:**
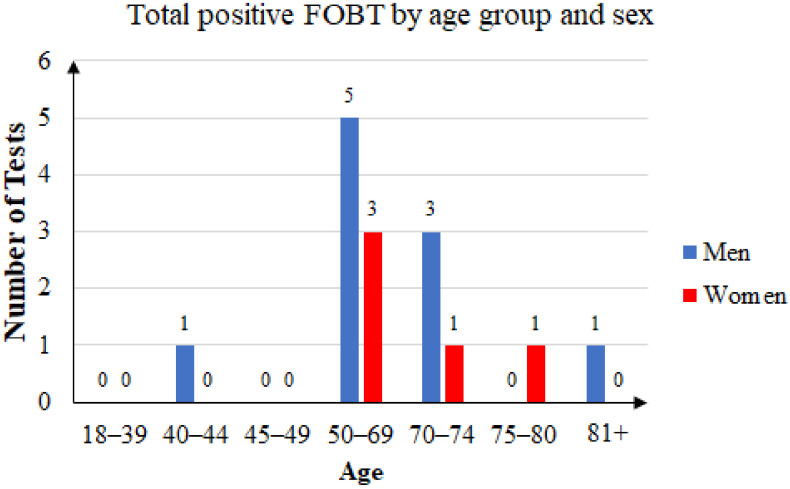
Total positive FOBT by age group and sex.

Of the 76 unique patients who came back with an FOBT result, 41 were women (53.95%) and 35 were men (46.05). Of those patients, 15 had a positive FOBT result (19.73%), of which 10 were men (66.67%) and 5 were women (33.33%). The age distribution is presented in Figure 6.

Of these 15 positive results, 3 were false positive results; only 12 patients remained with positive results. Table 2 presents the positivity rate of the FOB tests by age groups after removing the false positive results.

For 3 years, only six individuals, three men and three women, had a colonoscopy, giving a colonoscopy compliance rate of 50%. None of them were diagnosed with colorectal cancer. Three of them had polyps removed during a colonoscopy, but only two had a biopsy performed. Two of the patients had a colonoscopy performed with visualization of polyps, and the last was found with no polyps, only hemorrhoids.

## 4. Discussion

To the best of our knowledge, this is the first pilot colorectal cancer study attempted in Romania by a family doctor without any additional funding. It relied on the resources and pathways provided by the public healthcare system.

This study provided helpful information about engagement with the screening program and the obstacles that a Romanian patient needs to overcome to complete the screening when required. The patient with a positive FOBT result must navigate a tangled series of procedures to have a free colonoscopy in the public healthcare system. This procedure is available only during continuous or daily hospitalization. The availability and accessibility to being admitted to a hospital for this intervention are low. In many cases, patients opt to have a colonoscopy in the private healthcare sector just as an uninsured patient would. These costs are not excessive, but they represent a financial burden on a population that is already paying for healthcare services.

To test the hypothesis that it is not necessary to take an FOBT under 45 years of age or over 75 years, all patients above 18 years were asked to participate in the study [15]. We discovered one positive FOBT result in a 40-year-old patient, which was followed by a colonoscopy that detected a polyp, which was removed. Two 80- and 81-year-old patients were discovered with a positive FOBT result, but they refused a follow-up colonoscopy. The general feeling of the younger patients (under 45 years of age) was that they did not require screening because of hesitancy in providing stool samples and feeling that they were already healthy. They failed to understand the concept of prevention and showed signs of mistrust in government programs. This notion was made clear when vaccine hesitancy arose during the SARS-CoV-2 pandemic. The FOBT uptake rate was the lowest in their age group, at 33.33%. The older people had a sense of resignation, and they did not want to know for sure if they had colorectal cancer or not. No matter what their fate would bring, they accepted it. The FOBT uptake rate was the highest in their age group (over 75 years old), at 61.54%, but 0% when it came to taking a colonoscopy to complete the screening.

From the beginning, things did not run smoothly due to patients not understanding the importance of screening. For all of them, it was the first time they had heard about colorectal cancer screening. Not everybody was willing to participate in the study even though everything was free of charge. Due to limited open spots to perform no-cost tests in laboratories and the time required to have tests performed, some of the patients did not want to join the study. The primary reason is that Romania does not have dedicated screening pathways. All patients compete for the same number of tests that can be performed for free through the Romanian public health insurance system.

During the first period of the COVID-19 pandemic, we witnessed a paradox: large amounts of funds remained unused by laboratories because of people’s fear of going to places with potentially ill persons or because of lockdown. This is why we observed during the second year of the study a decrease in the number of patients enrolled. The rate of FOBT results was nearly 50%, the same as in the previous year. While the declining trend of new participants continued in the third year, we observed an increase in the number of patients that returned with FOBT results (67.74%).

Romanian authorities should have an important role in developing cancer awareness campaigns. Romania does not have a colorectal screening program financed by the government, but it has the promise of one [23]. On 3 November 2022, the Romanian Parliament adopted a law to prevent and combat cancer. The authorities should develop national cancer screenings according to this law [24].

We observed that women were more likely to adhere to screening than men, and the middle-aged and elderly were more likely to engage in screening than younger people. This is consistent with the publications on national programs for colorectal screening [21,25].

A further observation was that many of the patients who had agreed to be screened refused to participate after being advised to keep a 3-day diet before taking a stool sample. This led us to believe that if the test were FIT and not gFOB, this problem would have been solved. Four large-scale trials have examined gFOBT as a screening tool for CRC: Nottingham, U.K.; Funen, Denmark; Minnesota, U.S.; and Goteborg, Sweden [26,27,28]. The European guidelines for quality assurance in CRC screening and diagnosis concluded in 2010 that fecal immunochemical tests (FITs) offer enhanced analytical sensitivity and specificity and allow enhanced detection of both cancer and adenomas. Since these guidelines were published, all countries commencing population screening have adopted FIT [29].

Not every patient that agreed to participate in the screening program returned with an FOBT result. Some of them did not collect the stool sample; the majority of these were men. They had an important degree of adversity in taking the stool sample. The other group of patients that never returned with FOB results did take a stool sample and gave it to a laboratory. Unfortunately, the laboratory workers refused to perform the tests, arguing that the family doctor did not have legal authorization to recommend them. This is due to ever-changing legal recommendations when it comes to family medicine. The European consensus is granting more and more prerogatives to primary-care physicians, but healthcare workers are not always up-to-date with this information.

Because of the COVID-19 pandemic, patients with positive FOBT results could not proceed to a colonoscopy for a very long period. The lack of cancer screening also had a strong impact on cancer surgery on a global level. This caused a decrease in the occurrence of cancer diagnoses, particularly among asymptomatic patients [30,31]. When a colonoscopy was available, spots for a free one were scarce. This was because of the large number of serious cases that were not being taken care of during the lockdown. That is one of the reasons why screening should have dedicated pathways and not be mixed with the rest of the pathologies, competing for an open spot. Even so, the number of specialists that perform colonoscopies is low, and the availability of much-needed equipment is even lower.

We did not expect that patients would undergo a colonoscopy, and those who were discovered with one or two polyps would not have them removed during the colonoscopy. Another unfortunate occurrence was the fact that not all the patients had a biopsy performed.

We conclude that medical staff involved in screening need to be better educated and that medical resources (human and equipment) should be increased when a national CRC screening program is implemented in Romania.

The Bowel Cancer Screening Program (BCSP) in England invites subjects aged 56 to 74 to complete a fecal immunochemical test (FIT) every 2 years [32]. France also has a colorectal screening program that is addressed to a population aged from 50 to 74 years. This population receives an invitation to the test every 2 years [33]. Both countries have a two-step strategy. If the FIT is positive, a colonoscopy is performed. In Germany, an FIT can be carried out annually between the ages of 50 and 54 years and every two years from the age of 55. Starting at age 50 for men or 55 for women, the statutory screening program provides for a colonoscopy. If the screening is negative, a repeat colonoscopy can be performed after 10 years. As an alternative to a colonoscopy, a stool test is offered every two years [34,35]. The Slovenian National Colorectal Cancer Screening Program (SVIT) is aimed at men and women aged 50 to 74 years, who receive invitations for cooperation every two years. The primary screening test is a biennial fecal immunochemical test (FIT). All patients with positive tests are invited to undergo a colonoscopy [36,37]. The NordICC study (Northern European Initiative on Colon Cancer) suggests the benefits of colonoscopies for cancer screening may be overestimated [38]. This study has its limitations, and it is not a conclusion that we agree with.

Although there is no screening program at the national level for colorectal cancer, the ROCCAS and ROCCAS II programs were initiated in Romania following the EU Directive on colorectal cancer screening. These programs target underprivileged individuals, have dedicated pathways, and are funded by the European Union. They started in December 2018 and December 2019, respectively, and they aim to conduct screenings in pilot regions for the population aged 50 to 74. The following screening centers were nominated, according to well-defined criteria: the Fundeni Clinic Institute, the Central Military Emergency Clinic Hospital, the Constanța County Emergency Clinic Hospital, the Craiova County Emergency Clinic Hospital, and UMF Craiova, each of which has adjacent counties with a target of 50,000 people (total 200,000). The screened population receive immunological tests for the detection of fecal bleeding, and those positive will undergo a colonoscopy [23]. We have high expectations for these programs as they will reveal critical data about the behavior of underprivileged people.

### Study Limitations

Because this was a single-center pilot study with patients from mostly urban areas, the results may not accurately reflect the behavior of all patients, especially those from rural areas who are generally more compliant with their physicians’ advice. The COVID-19 pandemic limited access to other doctors, postponing the results. Repeat screenings within one year of the study were not considered. Not all eligible patients were invited to participate in the study because not all of them had reported to their GP during the study period. Participation in the program was linked to a visit to the family doctor for any health or administrative issues.

## 5. Conclusions

This pilot screening study reveals that there is a need for health education among Romanian patients, especially for prevention. By creating dedicated pathways for screening and providing patients with state-wide programs, Romanian health policymakers should play a more active role in this specific direction. Screening should be population-wide and not dependent on insurance. All healthcare workers involved need more applied training for improving communication skills regarding prevention and screening, updating to follow current protocols, and keeping up with legislative modifications. There should be no more cases in which patients are denied an FOBT in the public healthcare system on the sole basis that it was recommended by a GP. The delays in scheduling and performing colonoscopies indicate that significant numbers of specialists and equipment are needed.

## Figures and Tables

**Table 1:** FOBT uptake rate by age group.

Age Groups (Years)	Patients Enrolled	Patients with FOBT	FOBT Uptake Rate (%)
18–44	27	9	33.33
45–49	7	3	42.86
50–74	84	48	57.14
Over 75	26	16	61.54

**Table 2:** The positivity rate of FOB tests by age groups.

Age Groups (Years)	Total Number of FOB Tests	Positive FOB Tests	Positivity Rate (%)
18–44	9	1	11.11
45–49	3	0	0.00
50–74	48	9	18.75
Over 75	16	2	12.5

## Data Availability

Not applicable.
